# Investigation of the role of *MMP3 -1171insA* polymorphism in cutaneous malignant melanoma – a preliminary study

**DOI:** 10.1080/13102818.2014.947694

**Published:** 2014-11-13

**Authors:** Tatyana Vlaykova, Mateusz Kurzawski, Tanya Tacheva, Dimo Dimov, Maya Gulubova, Yovcho Yovchev, Stoyan Chakarov, Marek Drozdzik

**Affiliations:** ^a^ Department of Chemistry and Biochemistry, Medical Faculty, Trakia Univesity, Stara Zagora, Bulgaria; ^b^ Department of Experimental and Clinical Pharmacology, Pomeranian Medical University, Szczecin, Poland; ^c^ Department of Internal Medicine, Medical Faculty, Trakia Univesity, Stara Zagora, Bulgaria; ^d^ Department of General and Clinical Pathology, Medical Faculty, Trakia Univesity, Stara Zagora, Bulgaria; ^e^ Department of General Surgery, Medical Faculty, Trakia Univesity, Stara Zagora, Bulgaria; ^f^ Department of Biochemistry, Faculty of Biology, Sofia University, Sofia, Bulgaria

**Keywords:** skin malignant melanoma, MMP3, genetic predisposition, survival

## Abstract

Coetaneous malignant melanoma is the most aggressive cancer of the skin with a high rate of mortality worldwide. Degradation of basement membranes and extracellular matrix is an essential step in cancer invasion and metastasis. Matrix metalloproteinases (MMPs) and their tissue inhibitors (TIMPs) play key roles in this step. MMP-3 also called stromelysin-1 was one of the first proteinases found to be associated with cancer. In the gene of MMP-3 *(MMP3*), an insertion/deletion of an A nucleotide at position -1171 in promoter region has been identified and shown to effect the expression activity of the gene.

The present study was conducted to investigate the relation of *MMP3 -1171insA* polymorphism with skin malignant melanoma risk in a pilot case-control study of Bulgarian patients (n = 26) and unaffected controls (n = 172).

The genotypes of controls and melanoma patients were in Hardy-Weinberg equilibrium. The results showed no statistically significant difference both in genotype and allele frequencies of *MMP3 -1171insA* polymorphism between melanoma patients and healthy controls either in crude analyses (p = 0.360 and 0.790, c2-test) or after adjustment for age and sex. The comparison of some clinical characteristics between the patients with different genotypes showed a trend for longer survival of patients with *6A/6A* genotype compared to the carriers of *5A* allele (*5A/5A+5A/6A* genotypes, p = 0.118, Log rank test).

The results of our current preliminary study do not provide evidence for the role of the promoter polymorphism *-1171insA* in *MMP3* as a risk factor for development of coetaneous melanoma, but suggest its implication in progression of the diseases.

## Introduction

Malignant melanoma of the skin is a peculiar neoplasm with an unpredictable clinical course: it may remain silent for many years after its primary occurrence or it may behave in a very aggressive way and metastasize early.[1] Tumourogenesis in general, and melanoma development particularly, is a complex multi-step process accompanied by genetic and epigenetic changes which lead to acquisition of ability of cancer cells to invade the surrounding tissues and to disseminate into distant organs. These processes require enhancing of tumour angiogenesis and degradation of basement membranes and extracellular matrix, which are assisted by the increased expression and activity of matrix proteinases, such as plasminogen activators (t-PA and u-PA), cathepsins (cysteine or aspartyl proteinases) and matrix metalloproteinases (MMPs).[2]

MMPs are a large family of zinc-dependent natural endopeptidases that can degrade virtually all extracellular matrix components. At present, the family of MMPs consists of more than 20 members (currently, 23 in humans), which differ in substrate specificity, regulation and potential interactions with additional MMP and TIMP family members.[3–6] MMPs can be divided into the following five groups: collagenases (MMP-1, -8 and -13), stromelysins and stromelysin-like MMPs (MMP-3, -10, -11, -12), gelatinases (MMP-2 and -9), matrilysins (MMP-7 and -26) and membrane-type matrix metalloproteinases (MT-MMPs, MMP-14, -15, -16, -24, -17, -25).[6–10].

Gene expression of metalloproteinases is detected in particularly all cell types such as fibroblasts, keratinocytes, macrophages, endothelium cells, Langerhans dendritic cells, neurons, microglial cells, myocytes and in inflammatory infiltration cells (monocytes and T lymphocyte).[11] There is an abundant amount of evidence that MMPs and their endogenous TIMPs are over expressed in various tumour cells and tissues and pay key role in the process of cancer development and progression.[7,12,13]

The balance between MMPs and TIMPs is highly controlled at different levels and involves factors regulating the gene transcription, latent zymogene activation and inhibition by endogenous inhibitors.[7,14] It is proven that tumour cells can influence MMP expression either directly or by secreting soluble factors (extracellular matrix metalloproteinase inducer, EMMPRINs) that induce MMP in fibroblasts.[7,15] Most of the genes encoding MMPs and TIMPs respond to different stimuli at a transcriptional level due to the presence in their promoters of several functional *cis*-acting elements such as AP-1, Sp1, NFkB, RARE, Ets, STAT, Tcf/Lef, etc.[14]. Recently, the epigenetic regulation (methylation of CpG promoter islands, hypomethylation, histon acethylation) has been emerged as an important mechanism in balancing MMP/TIPM expression.[16] Moreover, the transcriptional activity of a variety of MMPs and TIMPs was found to be modulated by genetic polymorphisms in their promoter regions.[16]

MMP-3, also called stromelysin-1, was one of the first proteinases found to be associated with cancer. It can hydrolyse fibronectin, type IV, V, IX and X collagens, elastin, laminins, gelatin and proteoglycan core protein. It can also activate other proMMPs, including the collageneses MMP-1 and MMP-13.

MMP-3 has not been detected in ‘normal’ skin tissues distant from melanoma tumours, while high expression has been reported in the deep margins of melanoma and in the extracellular matrix (ECM) adjacent to the blood vessels, suggesting contribution of this enzyme in the processes associated with the invasiveness of malignant melanoma.[17,18] Moreover, earlier we found that high expression level of MMP-3 in melanoma metastases was associated with shorter disease-free survival.[19]

The gene of MMP-3 is located 11q23 in close proximity to *MMP1*. In *MMP3*, an insertion/deletion of an A nucleotide at position -1171 in the promoter region of *MMP3* has been identified. This promoter polymorphism (*5A/6A*, *-1171insA*, rs3025058) results in transcriptional activity of the *5A* homozygous in approximately double than the 6A homozygous.

So far in the current literature, there is only one study exploring the association of *MMP3 -1171insA* polymorphism with the risk of malignant melanoma.[20]

In this respect, the aim of the current pilot study was to identify *MMP3 -1171insA* genotype frequency and to evaluate its impact on the susceptibility to coetaneous malignant melanoma in a Bulgarian population from Stara Zagora region.

## Materials and methods

### Patients

The patient group consisted of 26 patients with coetaneous malignant melanoma, who were enrolled in the Oncology centre of Stara Zagora. Fifteen (58%) of the patients were males and the rest of them –11 (42%) were females, all aged between 42 and 77 years (median of 59.6 years). Nine (40.9) of the patients had pTNM stage I; seven (31.8%) had stage II, four (18.2%) had stage III and two (9.1%) had stage IV. Ten of the patients (45.5%, 10/22) with complete clinical records had developed metastases. The median disease-free survival of the patients, calculated from the date of diagnosis to the date of first appearance of metastasis, was 15.55 months (range of 0.00–125.81 months). The median overall survival of the patients (calculated from the date of diagnosis to the end of the follow-up period) was 32.82 months (range of 0.07–235.00 months), and as at the end of the following period (30.05.2013), 14 (58%) were dead and 10 (42%) alive.

The control group consisted of 172 healthy voluntaries or non-cancer hospital patients: 83 (48%) males and 89 (52%) females with an age ranging from 23 to 85 years (median of 61 years).

### Laboratory methods

Genomic DNA was isolated from 0.2 mL of whole blood using a commercial kit for isolation of genomic DNA from blood (GenElute™ Mammalian Genomic DNA Miniprep Kit, Sigma, USA). Determination of DNA concentration was performed spectrophotometrically.

The genotyping for *MMP3 -1171insA* (*5A/6A*, rs3025058) was performed by polymerase chain reaction – restriction fragment length polymorphism (OCR-RFLP)-based methods as it was described earlier by Vlaykova et al.[10] Restriction reactions for *MMP3 -1171insA* was carried out with 2U *Pdm I* (*Xmn I*) in final volume of 16 μL for 16 h at 37 °C. The fragments obtained after restriction reactions were analysed on 4% agarose gels. The gels were stained with ethidium bromide and documented with Gel documentation system (Syngene, Synoptics Ltd, UK). All experiments included known controls and blanks. About 5% of the samples were random selected and genotyping was reported for quality control.

### Statistical methods

Statistical analyses were performed using SPSS v16.0 (SPSS, Inc.). Survival curves were drawn with Kaplan–Mayer method and the difference in the survival was calculated with Log rank test. The genotype frequencies were tested for their fit to Hardy–Weinberg equilibrium. The odds ratio was calculated by using an interactive online software package http://statpages.org/#Package (http://statpages.org/ctab2×2.html). Factors with *p* < 0.05 were considered statistically significant.

## Results and discussion

**Figure 1.**
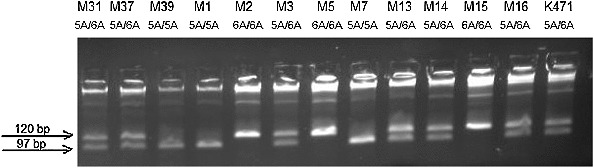
Agarose gel electrophoresis for genotyping for MMP3 *-1171insA* polymorphism.

The amplification with the primers for *MMP3* *-1171insA* resulted into 120 bp PCR product. *Pdm* I (*Xmn* I) digested the amplification product of the wide-type *5A* allele into two fragments (97bp and 23 bp), while the PCR product of the variant *6A* allele remained unchanged (one band of 120 bp) (Figure 1).

**Figure 2.**
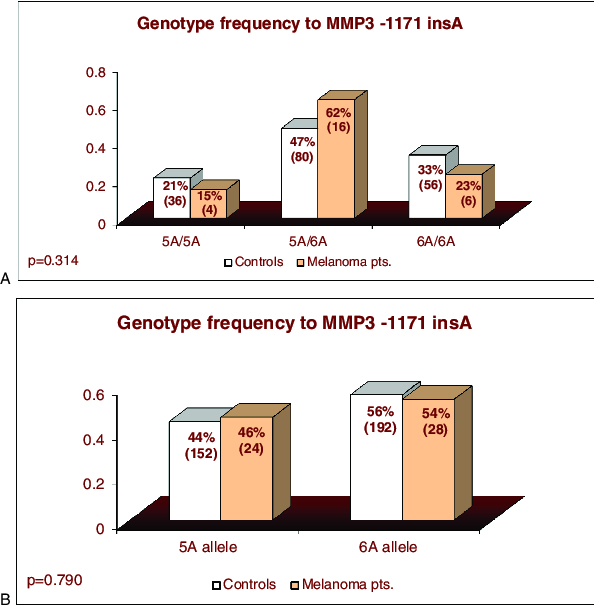
Distribution genotypes and alleles of *MMP3* *-1171insA* polymorphism in patients with coetaneous melanoma and in control individuals.

The results showed no statistically significant difference both in genotype and allele frequencies of *MMP3* *-1171insA* polymorphism between melanoma patients and healthy controls either in crude analyses (*p* = 0.360 and 0.790, *χ* ^2^-test) or after adjustment for age and sex (Figure 2(A) and 2(B) and Table 1)

**Table 1.** Genotype and allele distribution of MMP3 -*1171insA* in the groups of patients with coetaneous melanoma and controls and estimated ORs.

	Melanoma Pts.		Controls		
	*n*		*n*		
*MMP3* -*1171insA* (5A>6A)	*n* = 24	Frequency	*n* = 172	Frequency	OR (95% CI), *p*-value
Genotype frequency (crude analysis)					
Co-dominant model					
*5А*/*5А*	4	0.154	36	0.209	1.0 (referent)
*5А*/*6А*	16	0.615	80	0.465	1.80 (0.59–5.48) *p* = 0.429
*6А*/*6А*	6	0.231	56	0.326	0.96 (0.27–3.41) *p* = 0.957
Dominant model					
*5А*/*5А*	4	0.154	36	0.209	1.0 (referent)
(*5A*/*6A* + *6А*/*6А*)	22	0.846	136	0.791	1.46 (0.49–4.28), *p* = 0.512
Recessive model					
*5А*/*5А* + *5А*/*6А*	20	0.769	116	0.674	1 (referent)
*6А*/*6А*	6	0.231	56	0.326	0.62 (0.24–1.59), *p* = 0.331
Allele frequency					
-*1171 5А* (high-producing allele)	24	0.462	152	0.442	1.0 (referent)
-*1171 6А* (low-producing allele)	28	0.538	192	0.558	0.92 (0.52–1.65), *p* = 0.790

The carriers of genotypes with highly producing *5A* allele (*5А*/*5А + 5А*/*6А*) had 1.61-fold higher risk to develop skin melanoma; however, this result was not statistically significant (*p* = 0.320, Table 1).

**Figure 3.**
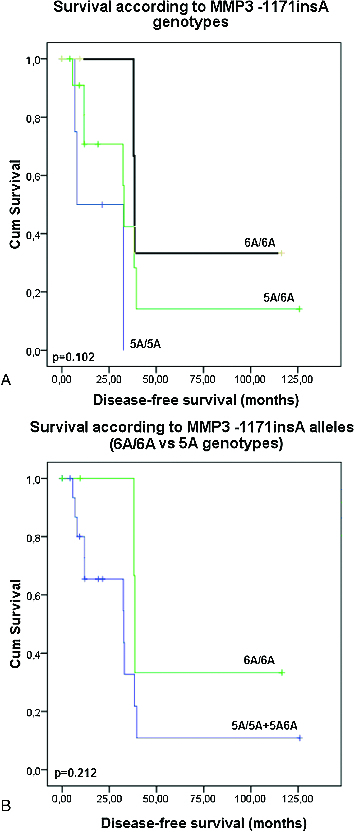
Disease-free survival of the patients with coetaneous melanoma according the *MMP3* *-1171insA* genotypes: (A) patients are divided in three groups – carriers of *5A/5A, 5A/6A* and *6A/6A* genotypes; (B) patients are divided into two groups: carriers of *5A* allele genotypes (*5A/5AC5A/6A*) and carriers of *6A/6A* genotype.

**Figure 4.**
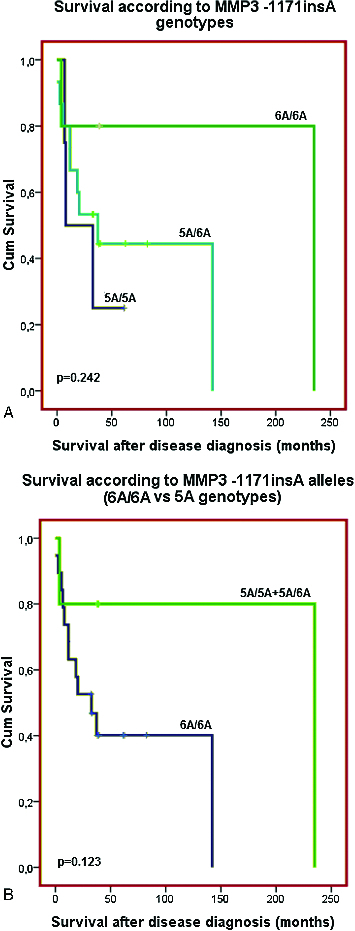
Overall survival of the patients with coetaneous melanoma according the *MMP3* *-1171insA* genotypes: (A) patients are divided in three groups – carriers of *5A/5A, 5A/6A* and *6A/6A* genotypes; (B) patients are divided into two groups: carriers of *5A* allele genotype (*5A/5AC5A/6A*) and carriers of *6A/6A* genotype.

No clinical or demographic characteristics were associated with the *MMP3 -1171insA* polymorphism. There was only a trend for longer disease-free survival (*p* = 0.101 and *p* = 0.212, Log rank test, Figure 3(A) and 3(B)) and overall survival (*p* = 0.242 and *p* = 0.123, Log rank test, Figure 4(A) and 4(B)) of those patients with *6A/6A* genotype compared to the carriers of 5A allele genotypes (*5A/5A C 5A/6A* genotypes, *p* = 0.123, Log rank test) (Figure 3(A) and 3(B)). The results of our current preliminary study do not provide evidence for the role of the promoter polymorphism *-1171insA* in *MMP3* as a risk factor for development of coetaneous melanoma. Similar lack of association between this polymorphism and risk of cancer was also reported from three large meta-analyses for digestive carcinoma,[21] lung cancer [22] and cancers with different origin.[23] Another meta-analysis of case-control studies of head and neck cancer (HNC) has also suggested that *MMP3* -*1171insA* polymorphism is not a risk factor in the overall patient population, but it is associated with HNC risk in some subgroups.[24] Based of our knowledge, the current preliminary study is the first one, evaluating the possible role of the *MMP3* -*1171insA* promoter polymorphism as risk factor for skin melanoma.

In the current literature, there is only one previous study exploring the association of *MMP3 -1171insA* polymorphism with malignant melanoma progression.[20] In that study, genotyping for the germline polymorphisms *-1171insA* in *MMP3* and *-1306C>T* and *-735C*>*T* in *MMP2* was performed in a group of 1002 melanoma patients. The conducted univariate and multivariate analyses and survival estimates did not find significant association between the genotypes and clinical, pathological and epidemiological variables.[20] Analogously, no association was found between the *MMP3 -1171 5A*>*6A* polymorphism and survival of patients with lung cancer from Spain.[25]

In our study, we also did not obtained significant associations of *MMP3 -1171insA* genotypes with the clinical or demographic melanoma characteristics; however, there was a clear trend for longer survival of the patients with *6A*/*6A* genotype compared to the carriers of *5A* allele genotypes (*5A*/*5A + 5A*/*6A*). Our results are in line with those reported for HCV-related hepatocellular carcinoma where *MMP3 5A* carriers had a significantly poorer prognosis than *MMP3* 6A homozygous.[23] In addition, in breast cancer the presence of 5A allele at the *MMP3* promoter region was suggested to represent an unfavourable prognostic feature associated with more invasive disease.[26]

Our results and those above mentioned could be explained with the functional effect of *6A* allele leading to lower promoter transcriptional activity and decreased production of the MMP-3,[27] which is generally implicated in aggressiveness of the tumours, particularly metastatic melanoma.[4,12,13,28] As a support of this notion are our previous findings for shorter disease-free survival of patients with advanced melanoma treated with combined chemoimmunotherapy having metastases with high expression level of MMP-3 detected by immunohistochemistry.[19] Moreover, Walker and Woolley observed immunostaining for MMP-3 at the deeper potentially invasive margins of primary skin melanoma, while there was no evidence of that enzyme protein in the normal skin tissue surrounding each of melanomas.[17] In addition, the MMP-3 serum levels of melanoma patients, although not significantly different from those of control individuals, were higher in those patients having tumours with more aggressive histological characteristics such as higher mitotic index and the presence of ulceration.[29,30]

## Conclusion

In conclusion, from the results of our preliminary study we may suggest that the promoter polymorphism *-1171insA* in *MMP3* does not contribute to risk of the occurrence of coetaneous melanoma; however, it may have an implication in progression of the diseases.

However, much research and larger case-control studies are warranted to confirm the possible role of *MMP3* promoter polymorphism as a prognostic factor for coetaneous melanoma.
